# Characterization of UDP-glycosyltransferase family members reveals how major flavonoid glycoside accumulates in the roots of *Scutellaria baicalensis*

**DOI:** 10.1186/s12864-022-08391-1

**Published:** 2022-03-02

**Authors:** Tianlin Pei, Mengxiao Yan, Tian Li, Xiaoqiang Li, Yijia Yin, Mengying Cui, Yumin Fang, Jie Liu, Yu Kong, Ping Xu, Qing Zhao

**Affiliations:** 1 Shanghai Key Laboratory of Plant Functional Genomics and Resources, Shanghai Chenshan Botanical Garden, Shanghai, 201602 China; 2 National Key Laboratory of Plant Molecular Genetics, CAS Center for Excellence in Molecular Plant Sciences, Institute of Plant Physiology and Ecology, Chinese Academy of Sciences, Shanghai, 200032 China; 3 Shanghai Center for Plant Stress Biology and Center of Excellence in Molecular Plant Sciences, Chinese Academy of Science, Shanghai, 201602 China

**Keywords:** *Scutellaria baicalensis*, Flavonoid glycosides, Baicalin, UDP glycosyltransferase

## Abstract

**Background:**

Flavonoid glycosides extracted from roots of *Scutellaria baicalensis* exhibit strong pharmaceutical antitumor, antioxidative, anti-inflammatory, and antiviral activities. UDP glycosyltransferase (UGT) family members are responsible for the transfer of a glycosyl moiety from UDP sugars to a wide range of acceptor flavonoids. Baicalin is the major flavonoid glycoside found in *S. baicalensis* roots, and its aglycone baicalein is synthesized from a specially evolved pathway that has been elucidated. However, it is necessary to carry out a genome-wide study of genes involved in 7-*O*-glucuronidation, the final biosynthesis step of baicalin, which might elucidate the relationship between the enzymes and the metabolic accumulation patterns in this medicinal plant.

**Results:**

We reported the phylogenetic analysis, tissue-specific expression, biochemical characterization and evolutionary analysis of glucosyltransferases (*SbUGTs*) and glucuronosyltransferases (*SbUGATs*) genes based on the recently released genome of *S. baicalensis*. A total of 124 *UGTs* were identified, and over one third of them were highly expressed in roots. In vitro enzyme assays showed that 6 SbUGTs could use UDP-glucose as a sugar donor and convert baicalein to oroxin A (baicalein 7-*O*-glucoside), while 4 SbUGATs used only UDP-glucuronic acid as the sugar donor and catalyzed baicalein to baicalin. *SbUGAT4* and *SbUGT2* are the most highly expressed *SbUGAT* and *SbUGT* genes in root tissues, respectively. Kinetic measurements revealed that SbUGAT4 had a lower *K*m value and higher *V*max/*K*m ratio to baicalein than those of SbUGT2. Furthermore, tandem duplication events were detected in *SbUGTs* and *SbUGATs*.

**Conclusions:**

This study demonstrated that glucosylation and glucuronidation are two major glycosylated decorations in the roots of *S. baicalensis*. Higher expression level and affinity to substrate of SbUGAT4, and expansion of this gene family contribute high accumulation of baicalin in the root of *S. baicalensis*.

**Supplementary Information:**

The online version contains supplementary material available at 10.1186/s12864-022-08391-1.

## Background

*Scutellaria baicalensis* Georgi is an important medicinal plant belonging to the Lamiaceae family. This plant is widely used in China and other Asian countries for the treatment of inflammation, diarrhea, lung and liver infections [1]. Extracts from *S. baicalensis* were recently reported to inhibit the growth of a range of cancer cells [2]. Flavones in the roots of *S. baicalensis* are the major bioactive compounds responsible for these bioactivities, such as baicalein, wogonin and their glycosides. These root-specific flavones lack a 4′-OH group on their B-rings and are synthesized from a new, specially evolved pathway [3]. These compounds contribute most specific health benefits in *S. baicalensis* and specifically promote the apoptosis of tumor cells without toxicity in healthy cells [4, 5]. Baicalein is also reported to repress the replication of COVID-19 virus by inhibiting its 3C-like protease [6, 7].

Approximately 100 flavones have been reported in *S. baicalensis*, and glycosylation contributes dramatically to the diversity of flavone structures [2]. Glycosylation is a normal decoration for flavones that often occurs at the end of their biosynthetic pathway. This pathway plays an important role in the stabilization and enhancement of the water solubility of flavones, leading to the regulation of bioactivity, and the storage and detoxification of xenobiotics in plants [8]. Compared to their aglycones, glycosides can be easily absorbed by the human body and have the potential to improve pharmacokinetic and pharmacodynamic profiles, which makes glycosylation a promising technology for drug discovery [9]. Due to the multiple hydroxyl groups on most parent flavone backbones, regiospecific biocatalytic reactions mediated by enzymatic synthesis may be a more suitable strategy than chemical synthesis [10].

*Glycosyltransferases* (*GTs*) belong to a large multigene family that encodes enzymes catalyzing the transfer of sugar moieties from activated donor molecules to specific acceptor molecules, including sugars, lipids, proteins, nucleic acids, antibiotics and other small molecules [11]. GTs are usually classified on the basis of amino acid sequence similarities [12], and 114 families of GTs have been identified to date (GT1–GT114, http://www.cazy.org/GlycosylTransferases.html). In plants, flavone glycosylation is generally catalyzed by family 1 GTs (GT1s), referred to as uridine 5′-diphosphate (UDP) GTs (UGTs), which transfer a glycosyl group from UDP sugars to a hydroxyl group of acceptor molecules. There were 120 and 224 UGTs found in *Arabidopsis thaliana* and *Oryza sativa*, respectively [13, 14]. UGTs can utilize highly divergent sugar donors, including UDP-glucose (UDP-Glc), UDP-galactose (UDP-Gal), UDP-glucuronic acid (UDP-GluA), UDP-xylose (UDP-Xyl), and UDP-rhamnose (UDP-Rha) [15], and sugar acceptors can be flavonoids, flavonols, dihydroflavonols, flavanones, isoflavonoids and anthocyanins [16, 17]. The major glycosylation site residues are the 3-, 5-, 7-, 4′-OH positions of flavonoid sugar acceptors [15, 18, 19]. UGTs contain the conserved domain [F/W]-x(2)-[Q/L]-x(2)-[L/I/V/M/Y/A]-[L/I/M/V]-x(4,6)-[L/V/G/A/C]-[L/V/F/Y/A/H/M]-[L/I/V/M/F]-[S/T/A/G/C/M]-[H/N/Q]-[S/T/A/G/C]-G-x(2)-[S/T/A/G]-x(3)-[S/T/A/G/L]-[L/I/V/M/F/A]-x(4,5)-[P/Q/R]-[L/I/V/M/T/A]-x(3)-[P/A]-x(2,3)-[D/E/S]-[Q/E/H/N/R], which is involved in binding to the UDP moiety of the sugar nucleotide [20]. This amino acid consensus sequence in plant UGTs is located in the C-terminus, which corresponds to the above signature motif, termed the plant secondary product glycosyltransferase (PSPG) box.

Baicalin is the 7-*O*-glucuronidated product of baicalein. The activity of a glucuronosyltransferase in *S. baicalensis* was first described in 2000 [21], and a glucosyltransferase from *S. baicalensis* was also characterized at the molecular level that converts baicalein to oroxin A [22]. However, it is still unclear whether other glucosyltransferase or glucuronosyltransferase members participate in flavone biosynthesis pathways and whether they play different roles in different compounds. With the availability of the *S. baicalensis* genome [23], it is necessary to carry out a genome-wide study of glucosyltransferases and glucuronosyltransferases in this medicinal plant and to elucidate the relationship between the enzymes and the metabolic accumulation patterns in the plant. Here, we identified and characterized the UGT family members of *S. baicalensis* by phylogenetic analysis, expression profiles and in vitro enzyme assays. Our results provide important clues for the biosynthesis of baicalin by using synthetic biology.

## Results

### Metabolomic analysis of glycosides in the roots of *S. baicalensis*

Flavone accumulation profiles were retrieved from metabolome data of *S. baicalensis* roots [24]. The results showed that 69 glycosides totally were found in *S. baicalensis* root tissues, with 62 *O*-glycosides and 7 *C*-glycosides (Additional file 1 Table S1). There were 33 flavonoid 7-*O*-glycosides, which included 17 glucose moieties, 8 glucuronic acids moieties, 3 rutinose moieties, 2 malonylglucose moieties, 1 galactose moiety, 1 rhamnose moiety, and 1 uncertain hexose moiety (Additional file 2 Fig. S1), indicating that glucosylation and glucuronidation were two major glycosylated decorations in the roots of *S. baicalensis*.

### Identification of *UGT* genes in *S. baicalensis*

Based on an HMMER search of the *S. baicalensis* genome sequence, we identified 130 *UGT* candidates using 122 *UGT* sequences from *A. thaliana* as queries. The relevant sequences and gene loci are provided in Additional file 1 Table S2. After the removal of redundant sequences, a total of 124 *UGT* genes were obtained. The protein length of the predicted UGTs ranged from 205 to 663 residues.

**Fig. 1:**
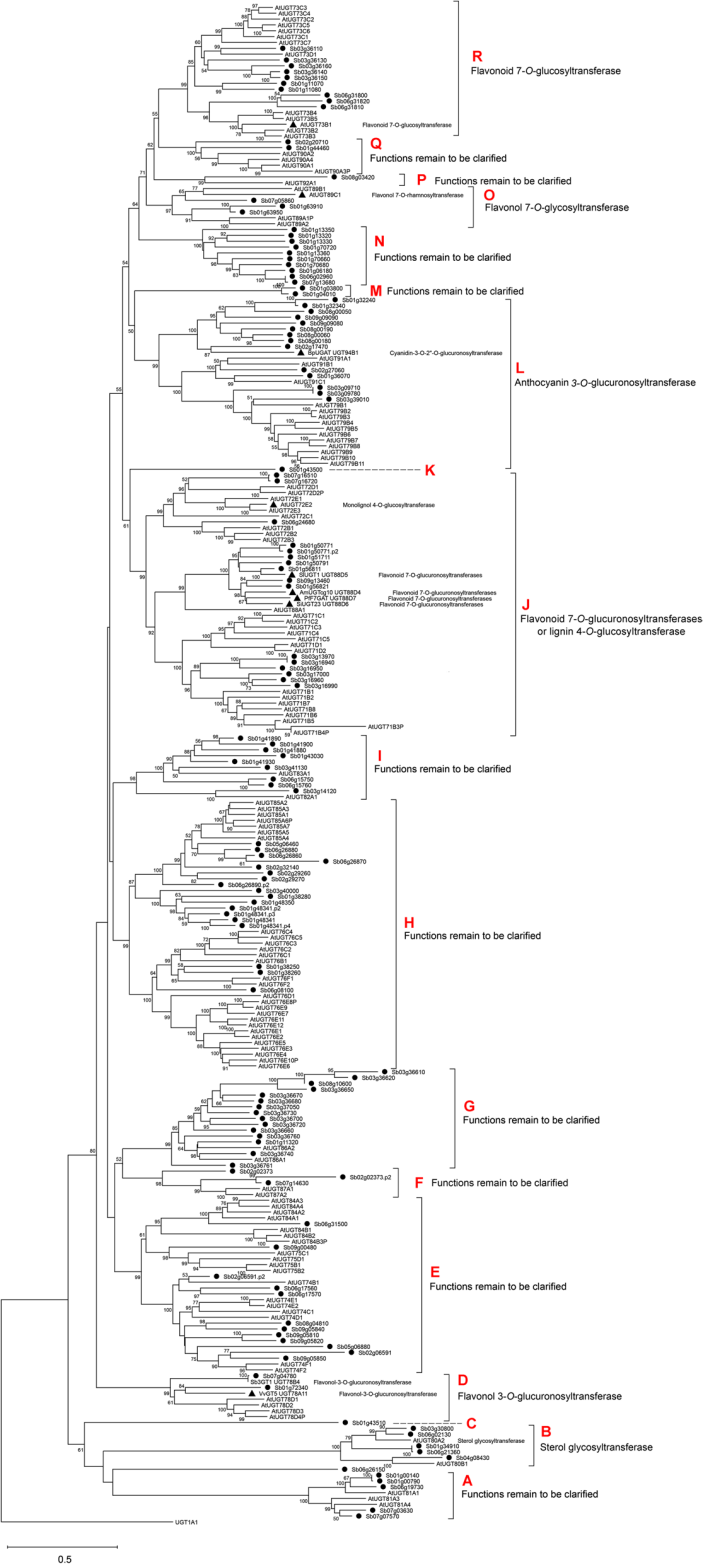
Phylogenetic tree of UGTs proteins. The neighbor-joining method was used to construct the tree with bootstrap (*n* = 1000). Circles before the labels represent candidate genes from *S. baicalensis*, triangles before the labels represent functional UGTs that have been reported. The postfix of the gene ID (.p2, .p3, .p4) represented different ORFs predicted for the same gene locus. UGT1A1 from *Homo sapiens* was used as an outgroup

The phylogenetic analysis of UGT sequences from *S. baicalensis* with those of *A. thaliana* and UGTs with known functions revealed that all candidates fell into 18 subfamilies (A to R) (Fig. 1). We annotated these subfamilies according to previous reports, including sterol glycosyltransferase (B) [25], flavonol 3-*O*-glucuronosyltransferase (D) [26], lignin 4-*O*-glucosyltransferase (J) [27], flavonoid 7-*O*-glucuronosyltransferase (J) [28], anthocyanin 3-*O*-glucuronosyltransferase (L) [29], flavonol 7-*O*-glycosyltransferase (O) [30] and flavonoid 7-*O*-glucosyltransferase (R) [31]. However, the functional classification of other subfamilies (A, C, E, F, G, H, I, K, M, N, P and Q) remains to be further studied. Moreover, subfamilies C and K contained only one *UGT* member from *S. baicalensis*, suggesting that these two genes encoding enzymes might catalyze the glycosylation of specific substrates.

### Chromosomal location of *UGT* genes in *S. baicalensis*

**Fig. 2:**
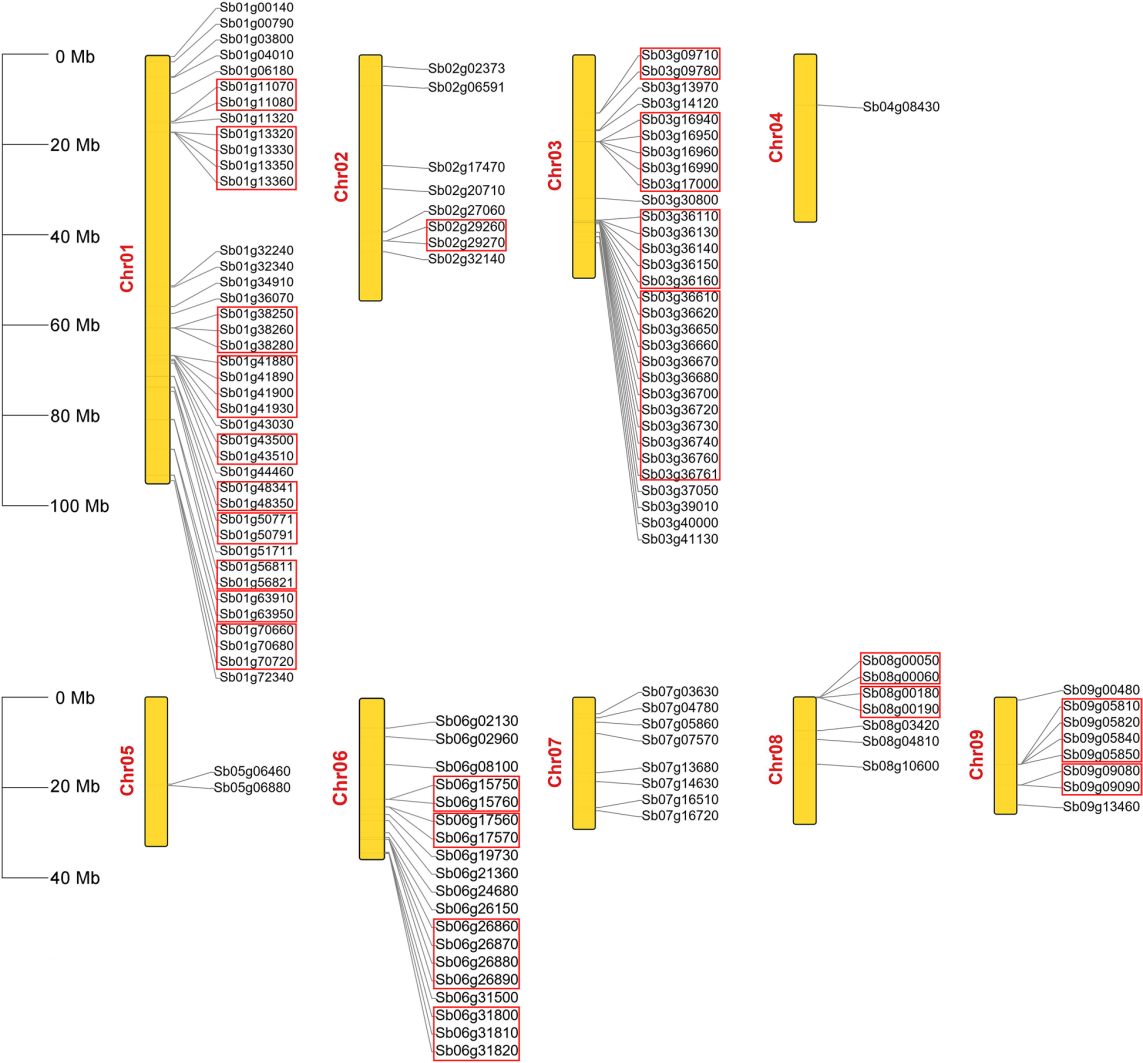
Chromosomal distribution of *UGT* genes in *S. baicalensis*. Yellow bars represent pseudochromosomes and red rectangles represent gene clusters

To detect *UGT* genes in the *S. baicalensis* genome in detail, we mapped the chromosomal localization of *UGTs* according to gene annotation files. As shown in Fig. 2, *UGT*s were unevenly distributed on all 9 pseudochromosomes (Chr01-Chr09) of *S. baicalensis*. Chr01 contained the most *UGTs* (40 genes), while Chr04 contained only one *UGT* gene. In addition, a gene cluster was used to describe the relative positions of *UGT* genes. According to the definition of gene clusters in which two or more genes fell within eight open reading frames (ORFs) on the same chromosome [32], 23 gene clusters covering two to twelve *UGT* genes were detected (Fig. 2). These gene clusters contained a total of 73 *UGT* genes which represented 58.87% of all *UGTs* located on chromosomes.

### Tissue-specific expression patterns of *UGT* genes in *S. baicalensis*

**Fig. 3:**
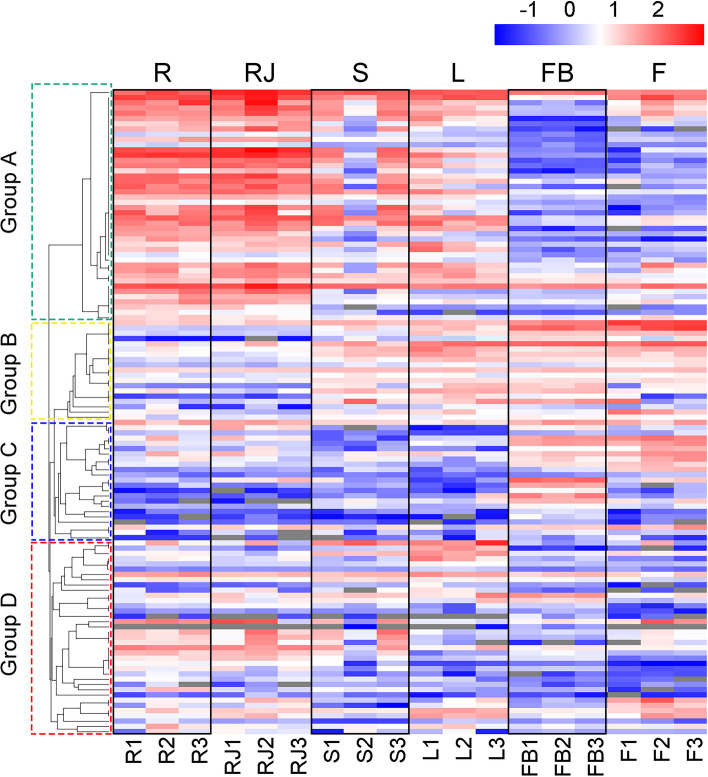
Tissue-specific expression heatmap of *UGTs* in *S. baicalensis*. The FPKM values of expression levels were normalized by log10, and the scale is shown at the top. R, root; RJ, root treated with MeJA; S, stem; L, leaf; FB, flower bud; F, flower; the numbers behind indicated the replicates. Rectangles with dashed line represent genes are clustered in different groups (group **A-D**) according to their expression patterns

Expression patterns of the *UGT* genes were analyzed using FPKM values from RNA-seq data of *S. baicalensis* [23]. Based on the expression patterns in different tissues, *UGTs* could be clustered into four main groups (Fig. 3). A total of 44 *UGTs* from group A, accounting for 35.5% of identified *UGTs*, had relatively high expression levels in roots, and most of them were induced by MeJA treatment, which indicated that these *UGT* family members were involved in the glycosylation of root-specific flavonoids in *S. baicalensis*. The expression levels of *UGTs* from group B were higher in the aerial parts (stems, leaves, flower buds and flowers) than in the roots, while members of group C were highly expressed in flower buds and flowers. The transcripts of *UGTs* from group D were equivalently distributed in all tissues examined.

### Gene isolation of 7-*O*-glycosyltransferases

Baicalin is the 7-*O* glucuronidated product of baicalein and is the richest flavonoid in *S. baicalensis* roots. To illustrate how major flavone glycoside accumulate, we identified 10 glucosyltransferases (*SbUGT1-10*) and 7 glucuronosyltransferase (*SbUGAT1-6*) candidate genes in subfamilies A and I (Fig. 1 and Additional file 1 Table S3), which might be involved in flavonoid 7-*O* glycosylation. SbUGAT1.1 and SbUGAT1.2 were two distinct ORFs predicted for the same gene locus. The full-length cDNAs of all the *SbUGTs* and *SbUGATs* were successfully isolated using specific primers (Additional file 1 Table S4). The genes were then reconstructed into a prokaryotic expression vector. Amino acid sequence alignment showed that the enzymes all possessed the conserved PSPG motif, and differences in two amino acids (Trp and Arg in SbUGAT) accounted for the functional divergence of UGT and UGAT (Additional file 2 Fig. S2) [28].

**Fig. 4:**
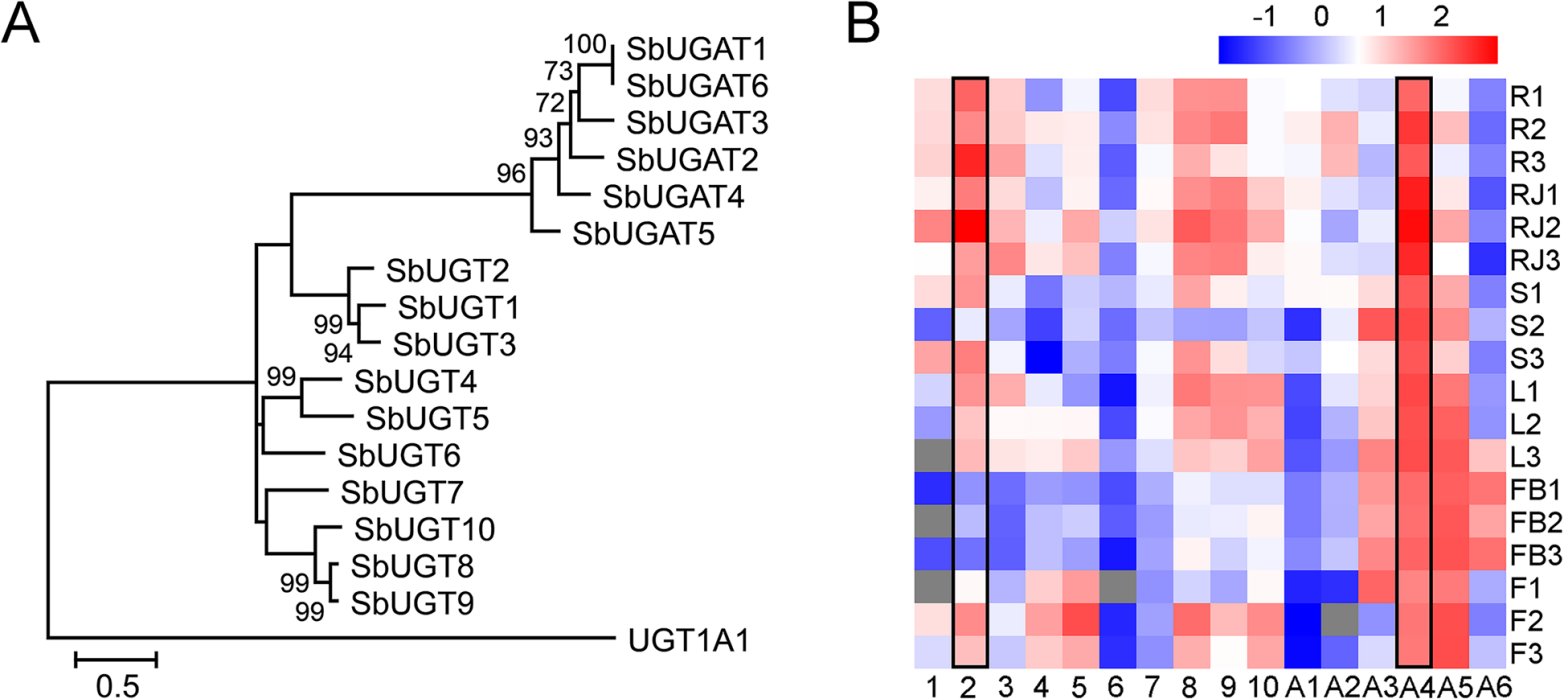
Phylogenetic analysis and expression patterns of *SbUGT* and *SbUGAT* genes. **A**. Phylogenetic tree of SbUGT and SbUGAT proteins. The maximum-likelihood method was used to construct the tree with bootstrap (*n* = 1000). UGT1A1 from *Homo sapiens* was used as an outgroup. **B**. Tissue-specific expression heatmap of *SbUGT* and *SbUGAT* genes. The FPKM values of expression levels were normalized by log10, and the scale is shown at the top. R, root; RJ, root treated with MeJA; S, stem; L, leaf; FB, flower bud; F, flower; the numbers behind indicated the replicates. Numbers on the bottom represent *SbUGT1* (1), *SbUGT2* (2), *SbUGT3* (3), *SbUGT4* (4), *SbUGT5* (5), *SbUGT6* (6), *SbUGT7* (7), *SbUGT8* (8), *SbUGT9* (9), *SbUGT10* (10), *SbUGAT1* (A1), *SbUGAT2* (A2), *SbUGAT3* (A3), *SbUGAT4* (A4), *SbUGAT5* (A5), and *SbUGAT6* (A6). The columns with black boxes represent the *SbUGT* and *SbUGAT* which had the highest FPKM values of expression levels in roots

The SbUGTs and SbUGATs were clearly separated in the phylogenetic tree (Fig. 4A). SbUGT1 clustered with SbUGT2 and 3, which was the sister group of SbUGATs clade. SbUGT4, 5 and 6 comprised a subgroup, while SbUGT7, 8, 9 and 10 clustered together. As shown in Fig. 4B, *SbUGT1*, *2*, *3*, *7*, *8*, and *9* and *SbUGAT1*, *2*, and *4* had relatively high expression in both roots and MeJA-induced roots, suggesting that these genes could be involved in the biosynthesis of flavonoid glycosides in roots. Transcripts of *SbUGT4*, *5*, *6*, *10*, and *SbUGAT3, 5* were highly accumulated in stems, leaves and flowers, while *SbUGAT6* seemed to be a flower bud specific gene, which was probably involved in decorations of flower pigments.

### Functional characterization of SbUGTs and SbUGATs

**Fig. 5:**
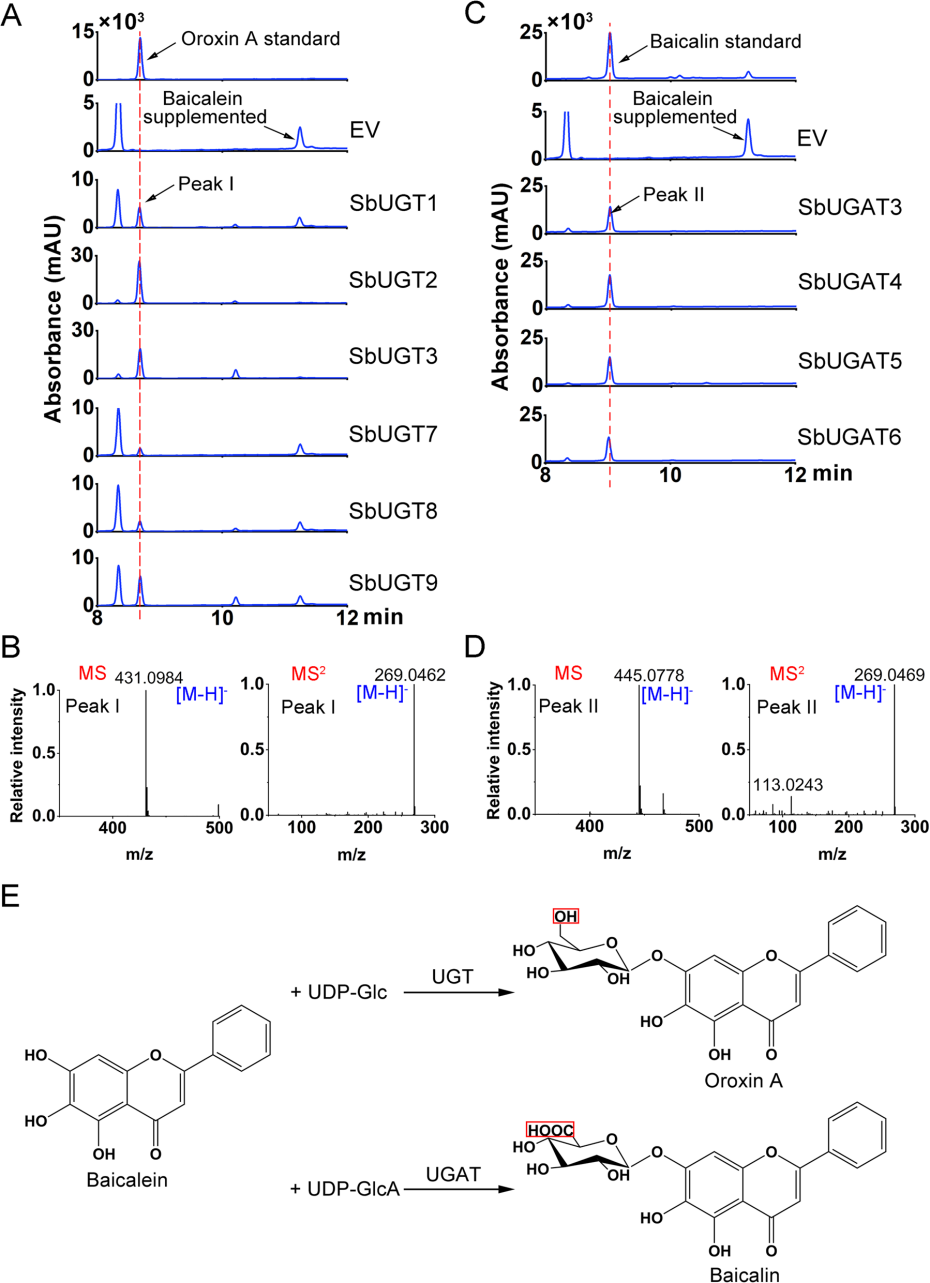
In vitro enzyme assays of SbUGTs and SbUGATs. **A**. HPLC analysis of SbUGTs using baicalein as a substrate in vitro enzyme assays. Top, oroxin A standard; EV, empty vector control; SbUGT1-3 and SbUGT7-9, assays with corresponding SbUGT proteins. **B**. MS and MS^2^ patterns of peak I products, which were identical to oroxin A standard. **C**. HPLC analysis of SbUGATs using baicalein as a substrate in vitro enzyme assays. Top, baicalin standard; EV, empty vector control; SbUGAT3-6, assays with corresponding SbUGAT proteins. **D**. MS and MS^2^ patterns of peak II products, which were identical to baicalin standard. **E**. The reaction catalyzed by SbUGT and SbUGAT using baicalein as a substrate. Red boxes indicated the different groups between sugar moieties

For in vitro enzyme assays, crude proteins of the candidate SbUGTs and SbUGATs were extracted from *Escherichia coli* carrying the corresponding genes, respectively. Compared with the empty vector (EV) control, new peaks (Peak I) with identical retention time to oroxin A standard were detected by HPLC from 6 SbUGT proteins (SbUGT1, SbUGT2, SbUGT3, SbUGT7, SbUGT8 and SbUGT9) incubated with baicalein as a substrate and UDP-Glc as a sugar donor (Fig. 5A). These products had the same mass charge ratio (m/z) and MS/MS patterns as oroxin A standard (Fig. 5B and Additional file 2 Fig. S4A). Correspondingly, new peaks (Peak II) were found by HPLC from 4 SbUGAT proteins (SbUGAT3, SbUGAT4, SbUGAT5 and SbUGAT6) supplemented with baicalein as a substrate and UDP-GluA as a sugar donor (Fig. 5C). The products were determined by comparing their retention time, m/z ratio and MS/MS patterns with baicalin standards (Fig. 5D and Additional file 2 Fig. S4B). These results indicated that SbUGTs could use UDP-Glc but not UDP-GluA as a sugar donor and convert baicalein to oroxin A, while SbUGATs used only UDP-GluA as the sugar donor and catalyzed baicalein to baicalin (Fig. 5E).

### Kinetic measurement of SbUGTs and SUGATs

The recombinant enzymes were purified from crude proteins that exhibited GT activity toward baicalein for kinetic analysis (Additional file 2 Fig. S4). As shown in Table 1 and Additional file 2 Fig. S5, SbUGT3 had the lowest *K*m (2.67 μM) among all the SbUGTs, but the highest *V*max value was detected for SbUGT2, which was 44,503 pkat mg^− 1^ protein, leading to a 3.36-fold higher *V*max/*K*m for SbUGT3 than for SbUGT2. However, SbUGT2 had the most abundant transcripts in roots compared with other SbUGTs, with an FPKM value 10.27 times that of SbUGT3. The lowest SbUGAT *K*m value was found for SbUGAT3, and SbUGAT6 had the highest *V*max/*K*m ratio. Furthermore, *SbUGAT4* was the most highly expressed *SbUGAT* gene in root tissues. Although *V*max/*K*m of SbUGAT6 is slightly higher than that of SbUGAT4, SbUGAT6 has few transcripts in roots and JA-treated roots, as its FPKM is 0.36 compared to 222.29 of SbUGAT4. In *S. baicalensis* roots, *SbUGT2* and *SbUGAT4* had the comparable expression levels, which were significantly higher than those of other *SbUGT* or *SbUGAT* genes. Therefore, competition between SbUGT2 and SbUGAT4 would determine the metabolic patterns in *S. baicalensis* roots. As SbUGAT4 had a higher *V*max/*K*m value for baicalein, being 1.4 times higher than that of SbUGT2, this explains the large amount accumulation of baicalin, rather than oroxin A, in *S. baicalensis* roots. SbUGT2 and the other SbUGTs might be involved in the biosynthesis of 4′-hydroxylated flavone 7-*O*-glucosides, such as luteolin 7-*O*-glucoside and apigenin 7-*O*-glucoside.

**Table 1:** Kinetic parameters and average FPKM values of roots and MeJA-induced roots of SbUGTs and SbUGATs

Enzymes	***K***m (μM)	***V***max (pkat mg protein^**− 1**^)	***V***max/***K***m	Average FPKM (Root)	Average FPKM (JA root)
**SbUBGT**					
SbUGT1	3.14	1027	327.07	12.53	28.18
**SbUGT2**	**100.60**	**44503**	**442.38**	**245.11**	**417.07**
SbUGT3	2.67	3964	1484.64	23.87	34.87
SbUGT7	–	–	–	8.73	6.93
SbUGT8	–	–	–	50.98	100.63
SbUGT9	–	–	–	53.69	86.67
**SbUBGAT**					
SbUGAT3	9.72	5607	576.85	2.38	2.63
**SbUGAT4**	**10.20**	**6266**	**614.31**	**222.29**	674.07 *
SbUGAT5	15.44	8161	528.56	10.05	16.68
SbUGAT6	12.32	7682	623.54	0.36	0.21

One asterisk (*) indicates a significant difference (0.01 < *P* < 0.05) between the root and MeJA-induced root under *t*-test. The products of SbUGT7, SbUGT8 and SbUGT9 were very low or not detected at the linear reaction stage

### Evolutionary analysis of *SbUGTs* and *SbUGATs*

**Fig. 6:**
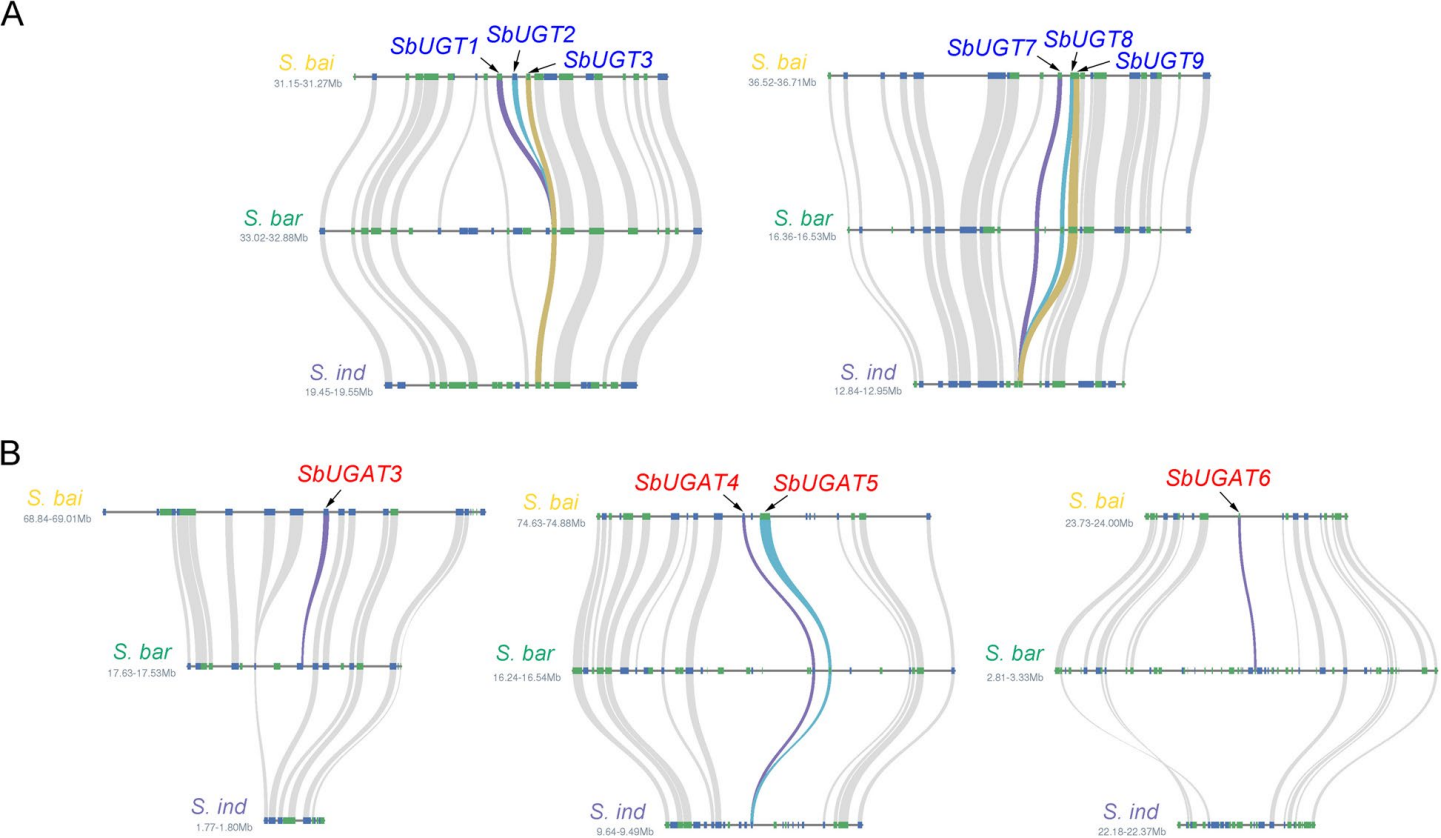
Evolutionary path of *SbUGTs* and *SbUGATs*. The syntenic relationships of *SbUGT* (**A**) and *SbUGAT* (**B**) genes were analyzed using the genome of *Scutellaria baicalensis* (*S. bai*), *Scutellaria barbata* (*S. bar*) and *Sesamum indicum* (*S. ind*). The highlighted lines indicate that syntenic genes can be found for *SbUGTs* or *SbUGATs* in *S. barbata* or *S. indicum*

Comparative genome analysis showed that 77 gene families expanded in *S. baicalensis*n, including the UGT gene family, which indicated the importance of glycosylation in *S. baicalensis* [23]. To identify the evolutionary path of *SbUGTs* and *SbUGATs*, gene syntenic relationships were analyzed between *S. baicalensis*, *Scutellaria barbata* (another medicinal plant from the genus *Scutellaria* accumulating baicalin) and *Sesamum indicum*. As shown in Fig. 6A, *SbUGT1* (*Sb01g31800*), *2* (*Sb01g31810*), and *3* (*Sb01g31820*) were located adjacently on pseudochromosome 1 and derived from one common ancestor. In *S. barbata* and *S. indicum* there is a single gene corresponding to *SbUGT1*, *2* and *3* in the region syntenic to *S. baicalensis*, indicating that the tandem duplication of *SbUGTs* followed the divergence of *S. baicalensis* from other species of the genus *Scutellaria* (< 13.28 Mya) [33]. Another tandem duplication event was detected in *SbUGT7* (*Sb03g36130*), *8* (*Sb03g36140*) and *9* (*Sb03g36150*), which were located close on pseudochromosome 3 (Fig. 6A). In *S. barbata*, there were three genes corresponding to *SbUGT7*, *8* and *9*, and only one single syntenic gene was found in *S. indicum*, indicating that *UGT* gene expansion occurred in the divergence of the Lamiaceae and Pedaliaceae families (< 46.9 Mya) [23].

*SbUGAT3* (*Sb01g51711*) and *6* (*Sb09g13460*) were located on pseudochromosomes 1 and 9, respectively, although we did not find homologs of these two genes in the isogenic regions of *S. indicum* (Fig. 6B). In *S. barbata* there is a single gene corresponding to *SbUGAT3* and *6* in the region syntenic to *S. baicalensis*, suggesting that *SbUGAT3* and *6* arose following the differentiation of the Lamiaceae and Pedaliaceae families (< 46.9 Mya) [23]. *SbUGAT4* (*Sb01g56811*) and *5* (*Sb01g56821*) were located closely on pseudochromosome 1 and had syntenic genes in the isogenic regions of *S. barbata,* which were derived from one common ancestor gene in *S. indicum*, indicating that a tandem duplication event occurred in *SbUGAT4* and *5* with the divergence of the Lamiaceae and Pedaliaceae families (< 46.9 Mya) [23]. These results revealed the importance of glycosylation in *S. baicalensis*, and indeed most of the flavonoids found in *S. baicalensis* can be glycosylated [2].

## Discussion

The glycosylation catalyzed by UGTs is important for the stabilization and enhancement of the water solubility of natural products. *UGT* genes are also involved in the regulation of metabolic homeostasis, deactivation/detoxification of xenobiotics, and biosynthesis, storage and transport properties of specialized metabolites [34]. UGTs occur as gene families in plant genomes. A wide range of identification of *UGT* genes from lower to higher plants showed that there existed at least one *UGT* gene in *Chlamydomonas reinhardtii*, which increased to 21 and 142 *UGT* genes in *Physcomitrella patens* and *Selaginella moellendorffii*, respectively, and a range of 56 to 242 *UGT* genes were identified in many vascular plants, suggesting that the expansion of *UGT* family occurred early in the land plant lineage and continued to expand at various rates among vascular plant lineages [34, 35]. In our study, we identified 124 nonredundant *UGTs* by searching the *S. baicalensis* genome. These genes encoding sequences could be divided into 18 subfamilies (Fig. 1), consistent with the phylogeny of a collection of 246 biochemically characterized UGT protein sequences [36]. Different types of flavone UGTs were located in five distinct subfamilies (D, J, L, O and R) that correlated with their respective substrate- and region-specificities. UGTs from other subfamilies may be involved in the sugar decoration of various specialized metabolites, such as terpenoids, phenolics and cytokinins [36], which need to be further clarified.

Extracts from *S.baicalensis* have strong antitumor, antiviral, anti-inflammatory, neuroprotective, and hepatoprotective activities owing to the rich flavonoid compounds [1]. Approximately 100 flavonoids were identified in *S. baicalensis*, and most of them were mainly found in the roots and were glycosylated [2]. Baicalin is the most abundant flavonoid glycoside accumulating in the roots, and its aglycone, baicalein, is a 4′-deoxyflavone that is synthesized from a root-specific pathway [3]. Newly developed genome sequencing technologies helped us elucidate the specially evolved pathway for baicalein [3, 23, 37]. Compared to the general flavonoid biosynthetic pathway, cinnamic acid is catalyzed by cinnamate-CoA ligase-like 7 (CLL-7), chalcone synthase 2 (CHS-2), and chalcone isomerase (CHI) to form pinocembrin, a flavanone without a 4′-OH group. Pinocembrin is then converted by a specialized isoform of flavone synthase II-2 (FNSII-2) to form chrysin, which serves as the founding precursor of 4′-deoxyflavones. Chrysin is then decorated by flavone 6-hydroxylase (F6H) to produce baicalein. For the final biosynthetic step of baicalin, more than one-third of *UGTs* were found to be highly expressed in roots and MeJA-treated roots (Fig. 3), consistent with the accumulated patterns of root-specific flavonoid glycosides, which were also induced by JA [3]. These *UGT* family members might contribute to the diversification of flavonoid glycosides in *S. baicalensis* roots, such as baicalin and oroxin A which possess the same aglycone but different sugar moieties, wogonoside and scutellarin, which are both 7-*O*-glucuronidations, or linarin and iridin with different aglycones and sugar groups (Additional file 1 Table S1). SbUGAT4 has a stronger expression level and higher affinity for baicalein, meaning that this enzyme should be able to compete effectively with SbUGTs for substrate in roots (Table 1), which makes baicalin high accumulation in the roots of *S. baicalensis* other than oroxin A.

UGT and UGAT have a broad range of substrate selectivity but narrow sugar donor adaptability. In addition to baicalein, SbUGT use UDP-Glc as an unique sugar donor convert wogonin to wogonoside, another 4′-deoxyflavone and its glycosides specifically accumulated in *S. baicalensis* roots [22]. SbUGT is also found to catalyze the 7-*O*-glucosylation of 4′-OH flavones, such as apigenin, scutellarein and kaempferol [22]. A UGAT protein purified from cultured cells of *S. baicalensis* exhibited 7-*O*-glucuronidated activity with UDP-GluA as a sugar donor in baicalein, wogonin and scutellarein, and showed lowest *K*m value and highest *V*max/*K*m ratio to baicalein [21]. Noguchi et al. found that the Trp residue (W) in the PSPG box of SbUGTs might be responsible for the better selectivity for the UDP-Glc donor, while the corresponding Arg residue (R) of SbUGATs plays a critical role in the interaction with the UDP-GluA sugar donor [28]. Homology modeling and site-directed mutagenesis analysis showed that these two key amino acid residues within the PSPG motif were vital for the substrate selectivity of *UGT* and *UGAT* because the cationic guanidinium moiety of R can be in close proximity to the anionic carboxylate of the glucuronic acid moiety of UDP-GluA. The *UGAT* gene is ubiquitous among Lamiales but cannot be found in *Arabidopsis* (Brassicaceae), suggesting that the functional differentiation of UGT and UGAT might occur locally in the lineage of specific plants [38]. However, a flavonoid 3-*O*-glycosyltransferase (Sb3GT1) from *S. baicalensis* was reported to accept five sugar donor (UDP-Glc/−Gal/−N-acetylglucosamine /−Xyl/−arabinose) to catalyze 3-*O*-glycosylation of 17 flavonols [15]. Molecular modeling revealed that the smaller side chains of G15 and P187 within Sb3GT1 offered a broader interspace which was critical for the sugar donor and substrate promiscuity. Sb3GT1 was clustered into subfamily D in our phylogenetic tree (Fig. 1), indicating that functional diversifications of UGTs were occurred following the specie evolution.

Gene duplications are one of the primary driving forces in the evolution of genomes and have contributed to the formation of specialized metabolites [39, 40]. For example, the second gene involved in the synthesis of baicalein, *SbCHS2*, likely underwent several duplications to produce five gene copies encoding identical or near-identical proteins. Similar gene amplifications were also detected in *SbFNSII-2*, *SbPFOMT* and *SbF8H*, suggesting that increasing the gene and protein dosages resulted in greater flux along the 4′-deoxyflavone biosynthetic pathway [23]. Tandem duplication events were found in *SbUGTs* and *SbUGATs* (Fig. 6). *SbUGT1*, *2* and *3* were likely the products of gene duplications after the divergence of *S. baicalensis* and *S. barbata* (< 13.28 Mya), while the expansion of *SbUGT7*, *8* and *9,* as well as *SbUGAT4* and *5*, occurred earlier when the Lamiaceae and Pedaliaceae families diverged (< 46.9 Mya). *SbUGAT3* and *6* were likely produced following the emergence of the *Scutellaria* genus due to the absence of any homologs of these two genes in the isogenic regions of *S. indicum*. The expansions of *SbUGTs* and *SbUGATs* are consistent with the abundant flavonoid glycosides accumulated in *S. baicalensis*, especially for baicalin, which might reflect changes involving our ancestors′ selection for species in *Scutellaria* genus with higher levels of 4′-deoxyflavones in their roots for use in traditional Chinese medicine (TCM).

## Conclusions

Specialized metabolites from plants are powerful weapons for humans when challenged by a pandemic [41], such as the COVID-19 virus, which has infected 240 million people and killed over 4.5 million people as we prepared this manuscript (https://covid19.who.int/). Baicalein from the roots of *S. baicalensis* exhibited excellent performance in suppressing the replication of COVID-19 virus [6, 7]. Baicalin is the 7-*O* glucuronidated product of baicalein converted by UGATs. The sugar moiety contributes to stronger absorptivity in the human intestine, and the absorbed baicalein can be released from baicalin by hydrolase in humans [42]. For the final biosynthetic step of baicalin, 6 SbUGTs and 4 SbUGATs cloned in this study showed 7-*O*-glucosylated and 7-*O*-glucuronidated activities to baicalein. Spatially high expression in roots and preferred to the substrate of SbUGAT4 make glucuronidation of baicalein more easily in the roots of *S. baicalensis*. Furthermore, the biosynthesis of baicalein directly from glucose in vitro has been achieved by *E.coli* fed-batch fermentation, and the production reached 214.1 mg/L [43]. Our results provide a toolkit for the biosynthesis of baicalin by using synthetic biology.

## Methods

### Plant materials

Root samples for metabolomic analysis were collected from 2-month-old and 2-year-old *S. baicalensis* plants maintained in Shanghai Chenshan Botanical Garden. Samples were ground into powder in liquid nitrogen and then freeze dried. Twenty milligram of each sample was suspended in 2 ml of 70% methanol and then extracted in an ultrasonic water bath for 2 h. After centrifugation at 12,000 g for 10 min, the supernatant was filtered through a 0.2 μm Millipore filter before metabolite analysis. Three biological replicates for each sample were collected.

Different tissues for RNA-seq were collected from 3-month-old *S. baicalensis* plants, including roots, stems, leaves, flower buds, flowers, and MeJA-induced roots (100 μM MeJA treatment for 24 h). All tissues were collected with three biological replicates.

### Widely-target metabolomic analysis

Samples were analyzed using a UPLC-ESI-MS/MS system (UPLC, SHIMADZU Nexera X2; MS, Applied Biosystems 4500 Q TRAP) as described previously [24]. Chromatographic separation was carried out on a Waters ACQUITY UPLC HSS T3 C18 column (1.8 μm, 2.1 mm × 100 mm). The flow rate of the mobile phase consisting of 0.04% (v/v) acetic acid in water (A), and 0.04% acetic acid (v/v) with acetonitrile was set to 0.35 ml/min. The gradient program was set with starting conditions of 5% B. Within 10 min, a linear gradient to 95% B was programmed, and a composition of 95% B was held for 1 min. Subsequently, a composition of 5% B was applied within 0.10 min and maintained for 2.9 min. The column oven was set to 40 °C; the injection volume was 2 μl.

Linear ion trap (LIT) and triple quadrupole (QQQ) scans were acquired on a QQQ-LIT mass spectrometer (Q TRAP), API 6500 Q TRAP UPLC/MS/MS System, equipped with an ESI Turbo Ion-Spray interface, operating in positive and negative ion modes and controlled by Analyst 1.6.3 software (AB Sciex). The ESI source operation parameters were as follows: ion source, turbo spray; source temperature, 550 °C; ion spray (IS) voltage, 5500 V (positive ion mode)/− 4500 V (negative ion mode); ion source gas I (GSI), gas II (GSII), and curtain gas (CUR), 50, 60, and 30.0 psi, respectively; and collision gas (CAD) level, high. Instrument tuning and mass calibration were performed with 10 and 100 μmol/L polypropylene glycol solutions in QQQ and LIT modes, respectively. QQQ scans were acquired as multiple reaction monitoring (MRM) experiments with collision gas (nitrogen) set to 5 psi. The declustering potential (DP) and collision energy (CE) for individual MRM transitions were optimized. A specific set of MRM transitions was monitored for each period according to the metabolites eluted within this period.

### Genome-wide identification of *SbUGT* and *SbUGAT* genes

A total of 122 UGT protein sequences of *A. thaliana* were downloaded from http://www.p450.kvl.dk/UGT.shtml#seqs and were selected to implement multiple sequence alignments by CLUSTAL X (V2.0) [44]. Then, the output alignment file was used to construct a hidden Markov model (HMM) profile by using the “hmmbuild” module within HMMER v3.0 [45]. The HMM profile was used to extract full-length UGT candidates from the *S. baicalensis* genome by the HMM algorithm (HMMER), filtering by a length between 200 and 600 amino acids. Finally, the UDPGT domain retrieved from Pfam PF00201 (http://pfam.xfam.org/) was used to check the validation of the final *UGT* genes.

Multiple sequence alignments and phylogenetic tree construction were performed using MEGA X [46]. For the neighbor-joining tree, candidates were constructed under the default parameters with UGT sequences from *A. thaliana*, and UGTs with known functions under the following accession numbers: BpUGAT (AB190262), SlUGT1 (AB362989), AmUGTcg10 (AB362988), PfUGT50 (AB362991), SiUGT23 (AB362990), VvGT5 (AB499074), and Sb3GT1 (MK577650). SbUGT and SbUGAT candidates could be screened according to the annotated function and classified subfamilies. A maximum-likelihood tree was constructed under the default parameters with sequences of 7-*O* SbUGT and SbUGAT candidates.

### Gene location visualization

The chromosomal location of the *SbUGT* and *SbUGAT* genes was determined using TBtools v1.098652 [47].

### Gene cloning

The complete ORFs of the *SbUGT* and *SbUGAT* genes were amplified by RT-PCR using the primers listed in Additional file 1 Table S4. cDNA templates were chosen according to the tissue-specific expression patterns of *SbUGT* and *SbUGAT* genes. The ORFs of *SbUGT1* and *SbUGT10* were obtained by de novo synthesis (GenScript, Nanjing, China). According to the manufacturer′s instructions, fragments were cloned into the entry vector pDONR207 and prokaryotic expression vector pYesdest17 using the Gateway BP Clonase II Enzyme Kit and LR Clonase II Enzyme Kit (Invitrogen, MA, USA), respectively.

### Crude enzyme extraction and protein purification

The successfully constructed vectors were transformed into *E. coli* Rosetta (DE3) competent cells (Weidi Biotech, Shanghai, China). After growing at 37 °C for 12 h, transformant colonies were initially grown in 10 ml of LB liquid medium with 100 μg/ml ampicillin at 37 °C and 180 rpm for approximately 12 h and then transferred to 200 ml of LB liquid medium with 100 μg/ml ampicillin at 37 °C in a shaking incubator until the OD_600_ reached 0.6-0.8. Isopropyl β-D-thiogalactopyranoside (IPTG) was added to a final concentration of 1 mM and cultured at 16 °C and 120 rpm for 16 h. pET28a (similar backbone with pYesdest17) was used as an empty vector control.

For crude enzyme extraction, *E. coli* cells were harvested by centrifugation at 12,000 rpm and then resuspended in 50 mM phosphate buffer (pH 8.0) that contained 0.5 mM phenylmethanesulfonylfluoride (PMSF), 300 mM NaCl, and 2 mM β- mercaptoethanol. High-pressure cell disruption equipment (Constant Systems, Northants, UK) was used to crush the *E. coli* cells. After centrifugation at 4 °C and 12,000 rpm for 20 min, approximately 10 ml of supernatant (crude protein) was collected. An equal volume of 60% glycerin was added to the supernatant for storage at − 80 °C.

For protein purification, *E. coli* cells were harvested by centrifugation at 12,000 rpm and then resuspended in 10 ml of buffer A [50 mM phosphate buffer (pH 8.0), 0.5 mM PMSF, 300 mM NaCl, 2 mM β-mercaptoethanol and 10 mM imidazole]. High-pressure cell disruption equipment (Constant Systems, Northants, UK) was used to crush the *E. coli* cells. After centrifugation at 4 °C and 12,000 rpm for 20 min, the supernatant was mixed with 1 ml of Ni–nitrilotriacetic acid (NTA) agarose (Qiagen, Germany) and stirred at 4 °C for 1 h. The mixture was packed into a column and washed three times at 4 °C with 5 ml of buffer B [50 mM phosphate buffer (pH 8.0), 0.5 mM PMSF, 300 mM NaCl, 2 mM β-mercaptoethanol and 20 mM imidazole]. The protein was eluted by 1 ml of buffer C [50 mM phosphate buffer (pH 8.0), 0.5 mM PMSF, 300 mM NaCl, 2 mM β-mercaptoethanol and 250 mM imidazole], and the imidazole was removed by Ultracel regenerated cellulose membrane (Millipore, MA, USA). Protein concentrations were determined using the Bradford method [48] and analyzed by 10% SDS-polyacrylamide gel electrophoresis.

### In vitro enzyme assays and kinetic studies

Crude enzyme assays were performed in a 100 μl reaction volume, which contained 100 mM Tris-HCl buffer (pH 7.0), 0.5 mM sugar donor (UDP-Glc or UDP-GluA), 5 μl of extracted protein and 100 μM substrate. The reaction was incubated for 2 h at 37 °C. Methanol was then added at a final concentration of 70% to quench the reaction. The reaction mixture was filtered with a 0.2 μm Millipore filter and analyzed by LC–MS.

For kinetics measurements, baicalein was used at concentrations ranging from 0.5 to 200 μM. The reaction time was reduced to 10 min. *K*m and *V*max values were calculated from the Eadie-Hofstee plot.

### Standard compounds

Baicalein and baicalin were purchased from Sigma-Aldrich (St. Louis, MO, USA), and oroxin A was purchased from Yuanye-Biotech (Shanghai, China). Baicalein was dissolved in dimethyl sulfoxide (DMSO), while baicalin and oroxin A were dissolved in methanol.

### Metabolite analyses

Metabolites were analyzed using an Agilent 1260 Infinity II HPLC (high-performance liquid chromatography) system. Chromatographic separation was carried out on a Phenomenex Luna C18 (2) column (100 mm × 2 mm 3 μ) with a guard column. The flow rate of the mobile phase consisting of 0.1% (v/v) formic acid in water (A) and 1:1 acetonitrile/MeOH + 0.1% formic acid (B) was set to 0.26 ml/min. The gradient program was as follows: 0-3 min, 20% B; 20 min, 50% B; 20-30 min, 50% B; 36 min, 30% B; 37 min, 20% B; and 37-43 min, 20% B. The detection wavelength was 280 nm. The injection volume was 20 μl and the column temperature was 35 °C. The products of enzyme assays were measured by comparing the area of the individual peaks with standard curves obtained from standard compounds.

LC–MS/MS was carried out by Thermo Q Exactive Plus. Chromatographic separation was carried out on a Phenomenex Luna C18 (2) column (100 mm × 2 mm 3 μ) using the same gradient described above. Mass spectra were acquired in negative ion modes with a heated ESI source, and the parameters were as follows: aus. Gas flow, 10 l/min; aus. Gas heater, 350 °C; sheath gas flow, 40 l/min; spray voltage, 3.5 kV; capillary temperature, 320 °C. For full-scan MS/data-dependent (ddMS^2^) analysis, spectra were recorded in the m/z range of 50–750 at a resolution of 17,500 with automatic gain control (AGC) targets of 1 × 10^6^ and 2 × 10.^5^

### Syntenic analysis

The genomes of *S. baicalensis*, *S. barbata* and *S. indicum* were compared using MCScan Toolkit v1.1 [49] implemented in Python. The genomes of *S. baicalensis* and *S. barbata* were downloaded from the National Genomics Data Center (https://bigd.big.ac.cn/gwh) with accession numbers GWHAOTC00000000 and GWHAOTP00000000, respectively, and the genome of *S. indicum* v1.0 was downloaded from National Center for Biotechnology Information (NCBI) under the BioProject PRJNA186669. Syntenic gene pairs were identified using an all-vs-all BLAST search using LAST [50], filtered to remove pairs with scores below 0.7, and clustered into syntenic blocks in MCScan. Microsynteny plots were constructed using MCScan.

## Supplementary Information

**Additional file 1: Table S1.** Flavonoid glycosides detected in the root metabolome. **Table S2.** Sequences of UGT genes identified from *S. baicalensis* genome. **Table S3.** The list of enzyme names, gene locus, and their subfamilies of predicted 7-O glycosyltransferases in *S. baicalensis*. **Table S4.** Primers used for the cloning of SbUGT and SbUGAT genes.

**Additional file 2: Figure S1.** Representative 7-*O* flavonoid glycosides detected from roots of *S. baicalensis*. Red boxes indicated the different groups between sugar moieties. **Figure S2.** Alignment of SbUGTs and SbUGATs protein sequences. The consensus sequences were highlighted by red color. The arrows indicated the different amino acid residues between SbUGTs and SbUGATs, which were responsible for the functional divergent between these two types of glycosyltransferases. **Figure S3.** MS and MS^2^ patterns of oroxin A (A) and baicalin standard (B). **Figure S4.** SDS PAGE analysis of purification of SbUGT and SbUGAT proteins. A. Tracks from left to right showed protein markers (M), empty vector control (1), SbUGT1 (2), SbUGT2 (3), SbUGT3 (4), SbUGT7 (5), SbUGT8 (6) and SbUGT9 (7). B. Tracks from left to right showed protein markers (M), empty vector control (1), SbUGTA3 (2), SbUGAT4 (3), SbUGAT5 (4) and SbUGAT6 (5). **Figure S5.** Nonlinear regressions of the Michaelis−Menten equation for SbUGTs and SbUGATs.

## Data Availability

The DNA and the protein sequences from *S. baicalensis* are provided in Additional file 1 Table S2. Protein sequences from *A. thaliana* are available with the link of http://www.p450.kvl.dk/UGT.shtml#seqs. RNA sequencing data are available in the Sequence Read Archive (SRA) database with the link of www.ncbi.nlm.nih.gov/sra, under the accession number SRP156996. The genome of *S. baicalensis* and *S. barbata* are available in the National Genomics Data Center (https://bigd.big.ac.cn/gwh) with accession number GWHAOTC00000000 and GWHAOTP00000000, respectively, and the genome of *S. indicum* v1.0 is available in the National Center for Biotechnology Information (NCBI) under the BioProject PRJNA186669. The metabolome datasets and LC-MS profiles analyzed during the current study are not publicly available due competing interests but are available from the corresponding author on reasonable request.
