# Supplementary material for: Characterization of UDP-glycosyltransferase family members reveals how major flavonoid glycoside accumulates in the roots of Scutellaria baicalensis
Source: BMC Genomics. 2022 Mar 2;23:169. doi: 10.1186/s12864-022-08391-1 (PMC8888134; doi:10.1186/s12864-022-08391-1)
Supplement: Supplementary file 2 — Additional file 2: Figure S1. Representative 7-O flavonoid glycosides detected from roots of S. baicalensis. Red boxes indicated the different groups between sugar moieties. Figure S2. Alignment of SbUGTs and SbUGATs protein sequences. The consensus sequences were highlighted by red color. The arrows indicated the different amino acid residues between SbUGTs and SbUGATs, which were responsible for the functional divergent between these two types of glycosyltransferases. Figure S3. MS and MS2 patterns of oroxin A (A) and baicalin standard (B). Figure S4. SDS PAGE analysis of purification of SbUGT and SbUGAT proteins. A. Tracks from left to right showed protein markers (M), empty vector control (1), SbUGT1 (2), SbUGT2 (3), SbUGT3 (4), SbUGT7 (5), SbUGT8 (6) and SbUGT9 (7). B. Tracks from left to right showed protein markers (M), empty vector control (1), SbUGTA3 (2), SbUGAT4 (3), SbUGAT5 (4) and SbUGAT6 (5). Figure S5. Nonlinear regressions of the Michaelis−Menten equation for SbUGTs and SbUGATs. [file 12864_2022_8391_MOESM2_ESM.docx]

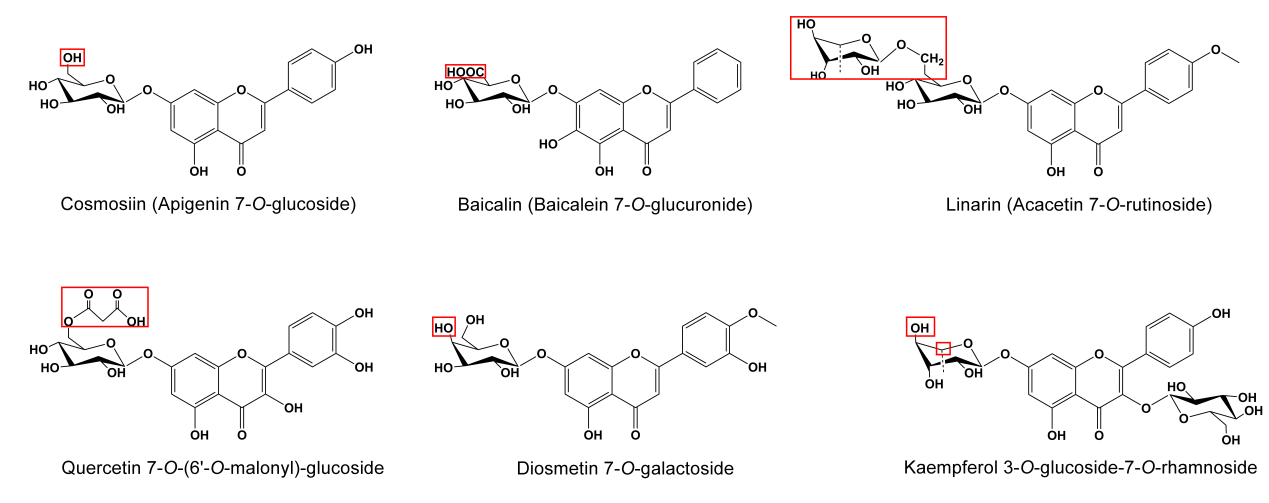


**Figure S1. Representative 7-*O* flavonoid glycosides detected from roots of *S. baicalensis***

Red boxes indicated the different groups between sugar moieties.


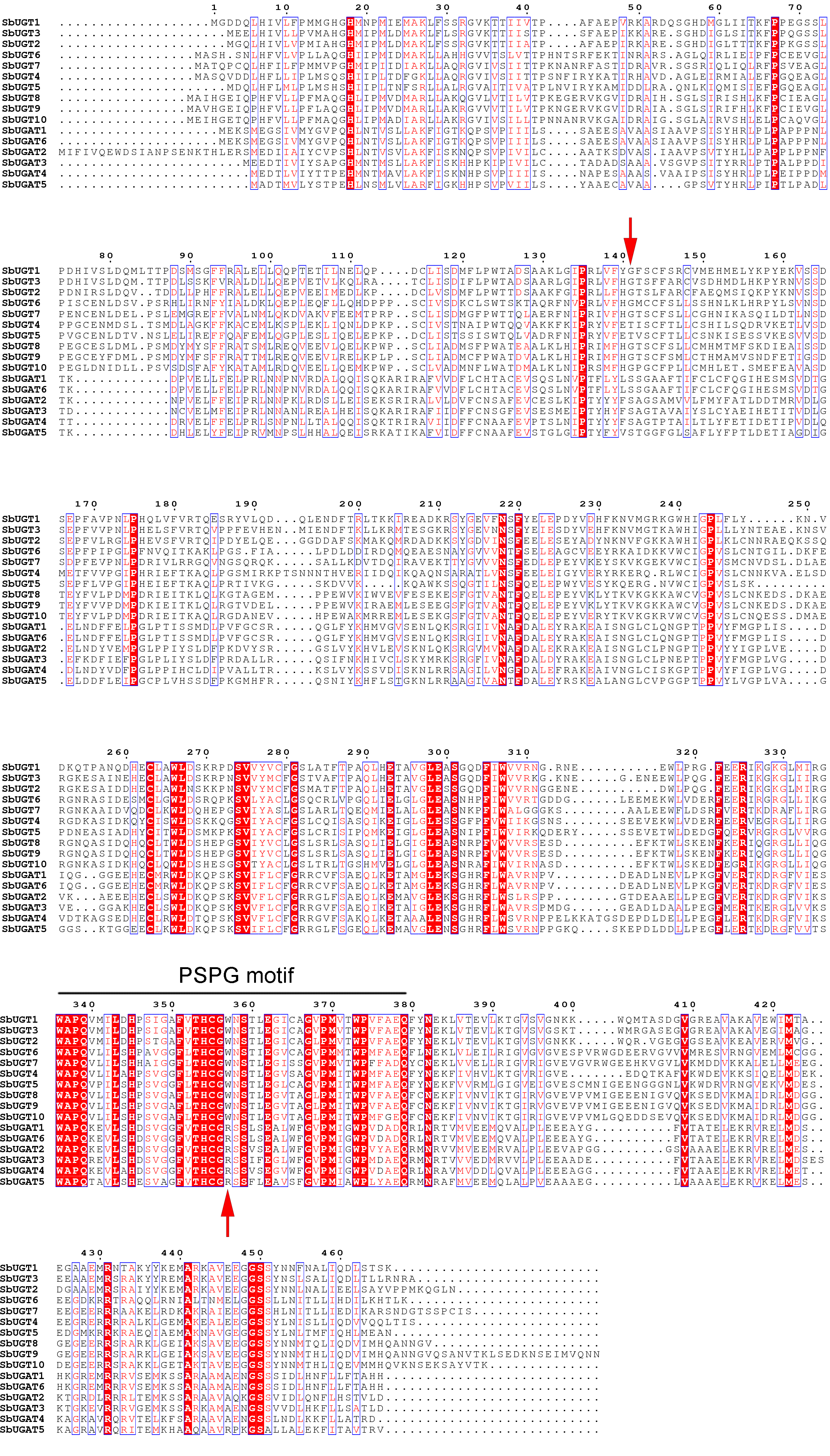


**Figure S2. Alignment of SbUGTs and SbUGATs protein sequences**

The consensus sequences were highlighted by red color. The arrows indicated the different amino acid residues between SbUGTs and SbUGATs, which were responsible for the functional divergent between these two types of glycosyltransferases.


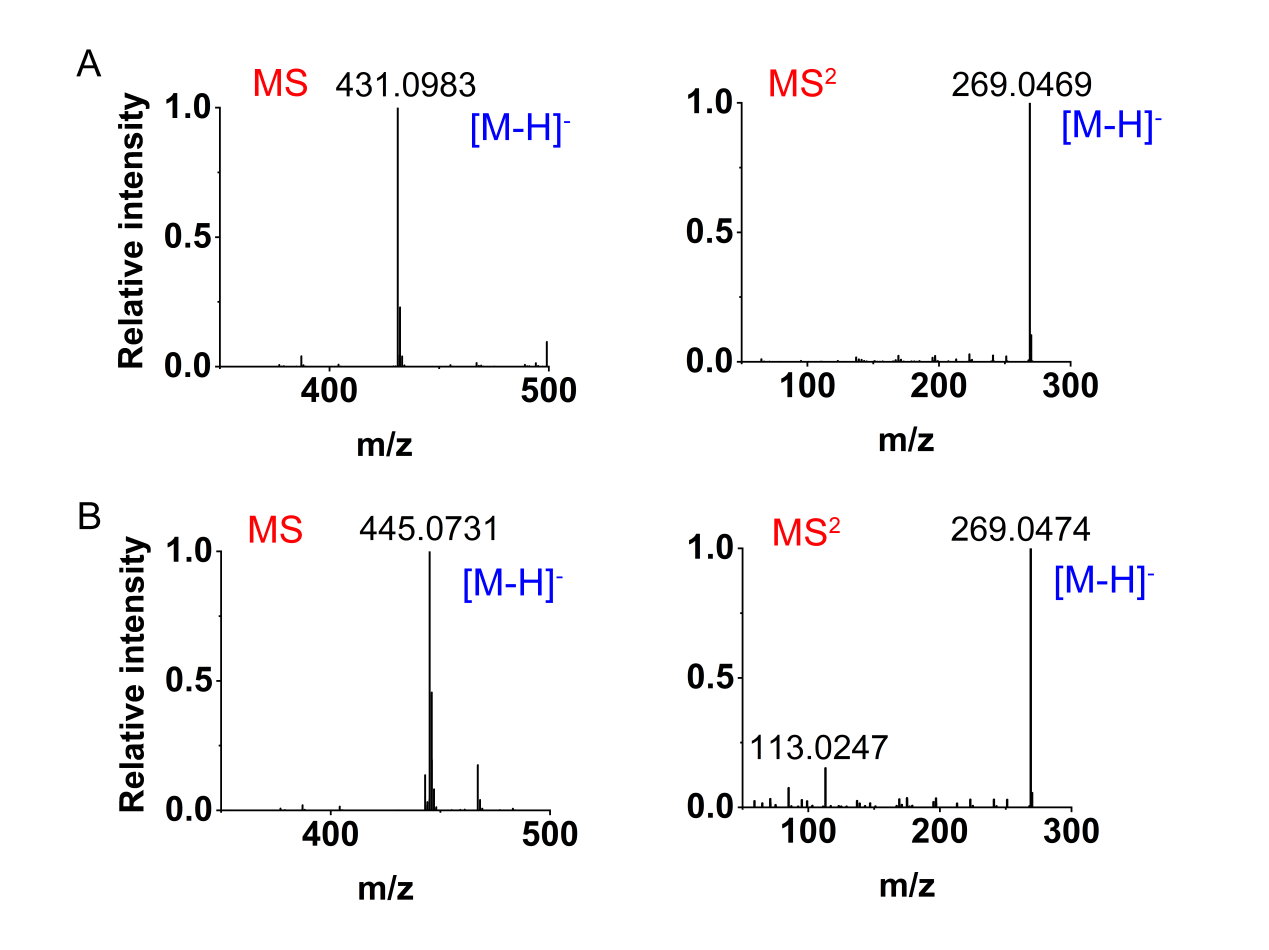
**Figure S3. MS and MS^2^ patterns of oroxin A (A) and baicalin standard (B).**

**
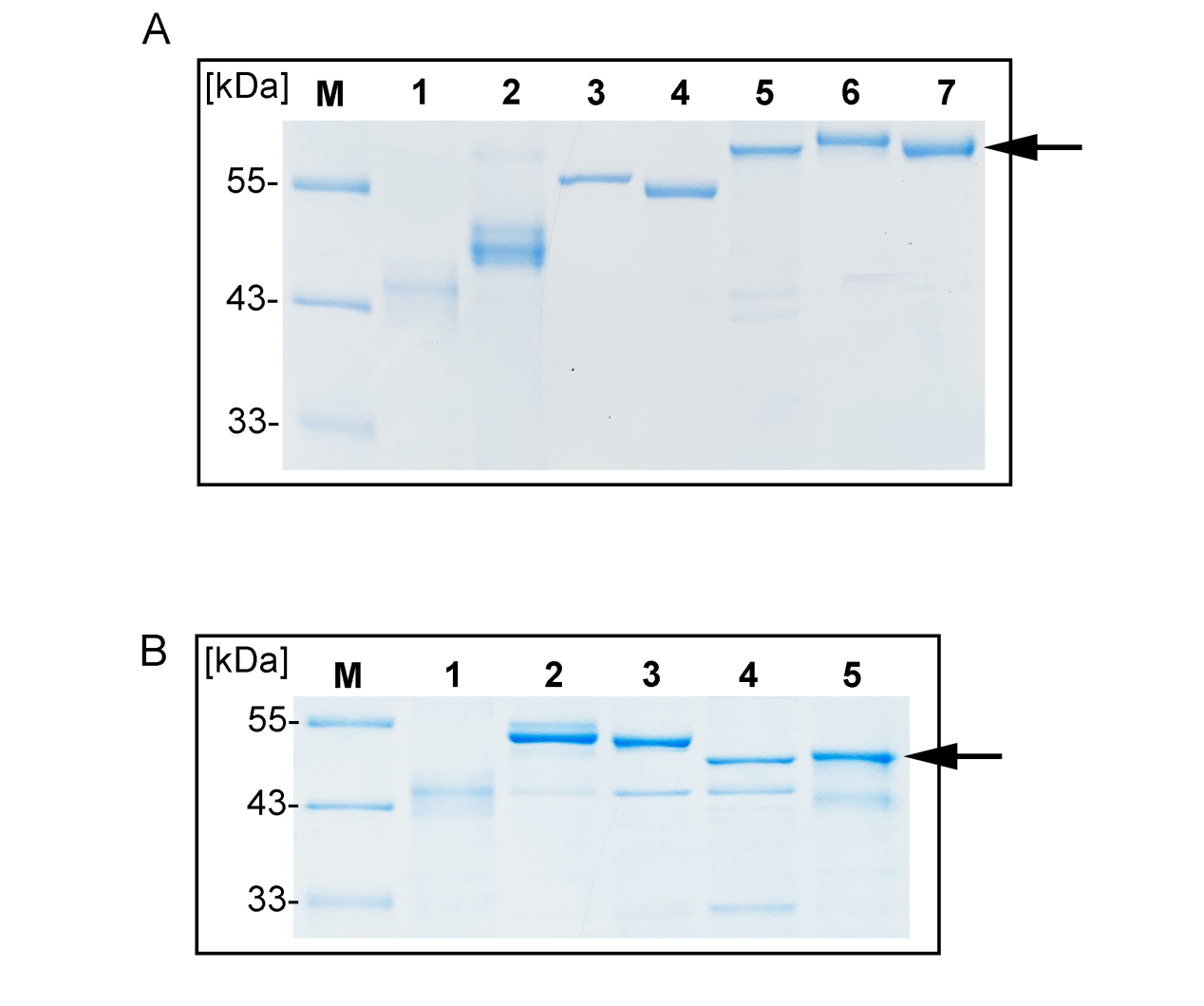
**

**Figure S4. SDS PAGE analysis of purification of SbUGT and SbUGAT proteins.**

A. Tracks from left to right showed protein markers (M), empty vector control (1), SbUGT1 (2), SbUGT2 (3), SbUGT3 (4), SbUGT7 (5), SbUGT8 (6) and SbUGT9 (7).

B. Tracks from left to right showed protein markers (M), empty vector control (1), SbUGTA3 (2), SbUGAT4 (3), SbUGAT5 (4) and SbUGAT6 (5).


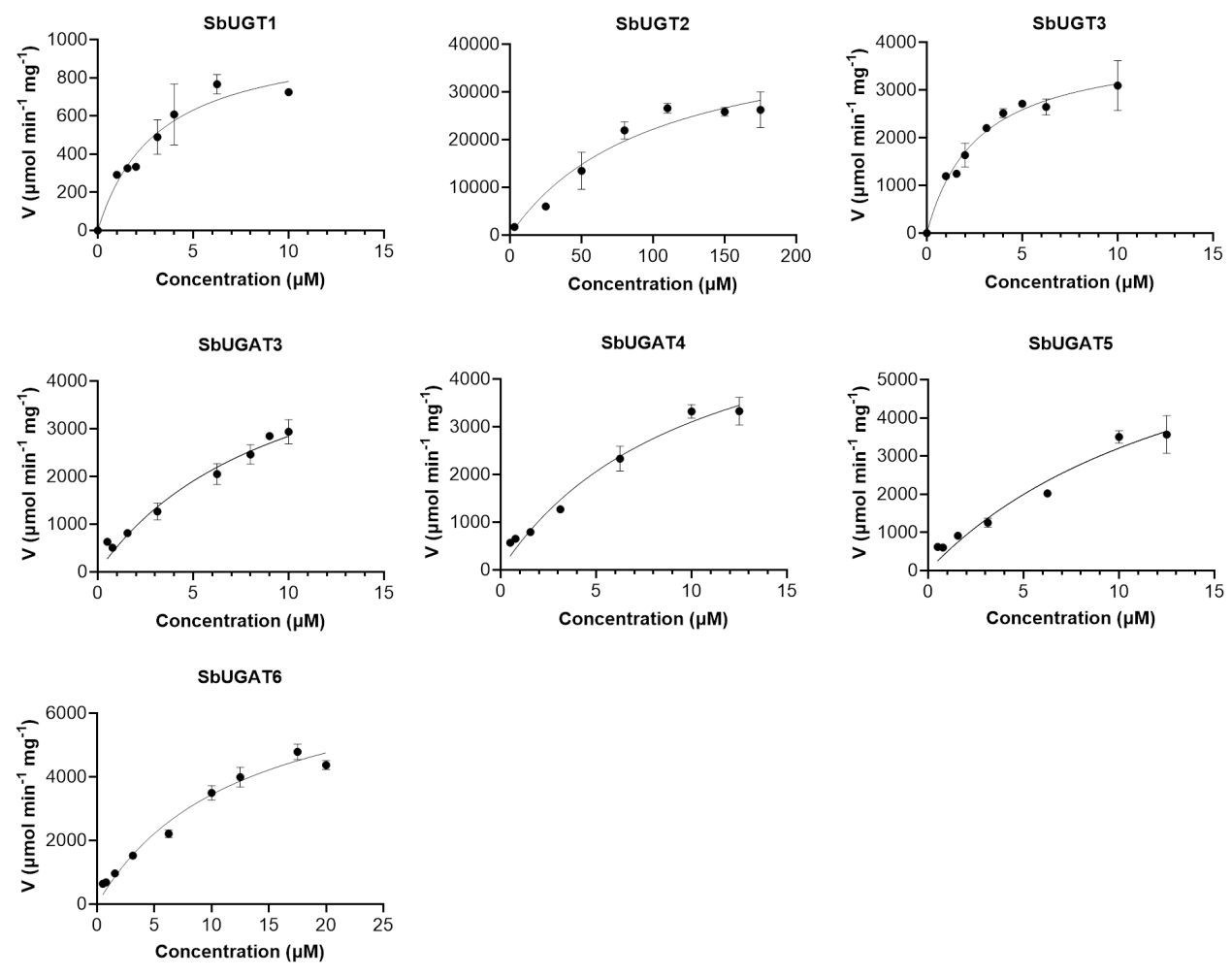


**Figure S5. Nonlinear regressions of the Michaelis−Menten equation for SbUGTs and SbUGATs**
